# University students’ awareness of the social dimension of sustainable transportation: The case of Amasya/Turkey

**DOI:** 10.1371/journal.pone.0346371

**Published:** 2026-04-28

**Authors:** Sedef Şendoğdu, Ceren Nergiz

**Affiliations:** 1 Department of City and Regional Planning, Faculty of Architecture, Ondokuz Mayıs University, Samsun, Türkiye; 2 Graduate School of Postgraduate Education, Department of City and Regional Planning, OMÜ Kurupelit Kampüsü Institute of Graduate Studies Atakum, Samsun, Türkiye; University of Jaen: Universidad de Jaen, SPAIN

## Abstract

Promoting walking and cycling is crucial for enhancing quality of life and supporting sustainable mobility. Although the literature includes numerous studies addressing the physical and environmental dimensions of sustainable transportation, the social dimension—particularly in relation to bicycle use—has not been sufficiently explored. This gap limits the wider adoption of cycling as a mode of transport. The present study examines university students’ awareness of the social dimension of sustainability, which represents a critical factor in advancing sustainable transportation. Amasya University in Turkey was selected as the case study area due to its diverse academic faculties, the existence of bicycle paths, and the suitability of its urban structure for cycling accessibility. The study aims to reveal how the awareness levels of students from different academic disciplines shape the development of social sustainability in transportation. A mixed-methods approach was adopted, combining attitude analysis, survey applications, and field observations. Attitude analysis was employed to evaluate individuals’ perceptions and behaviors and to explore the extent to which these tendencies can be influenced. The surveys and field observations enabled an integrated evaluation of both quantitative and qualitative data. The findings reveal significant differences in sustainable mobility awareness according to faculty affiliation and socio-demographic characteristics. Female students reported higher bicycle use (60%), while most cyclists belonged to middle- and low-income groups (73.2%). Approximately 47.3% of participants used bicycles for short distances, and 77.4% stated that they cycled primarily for health benefits. In addition, socio-cultural pressures influenced cycling behavior, as 80% of Theology students reported avoiding cycling due to social norms. Overall, the results indicate that indicators such as equity, accessibility, health and safety, individual responsibility, integrated planning, and cultural values and habits play significant roles in shaping students’ awareness and influencing their bicycle use. The study highlights the importance of integrating social dimensions into sustainable transportation policies and university education programs. By doing so, it provides both academic and practical insights for promoting bicycle use and supporting more inclusive and sustainable urban mobility systems.

## 1. Introduction

Throughout history, transportation systems have played a fundamental role in shaping and developing cities. However, the increasing urban population and expanding urban areas have made it imperative to address transportation needs within the framework of sustainability [1–6]. Defined as “meeting current transportation needs without compromising the rights of future generations,” sustainable transportation consists of three main dimensions: economic, environmental, and social sustainability [7–9].

In recent years, sustainable mobility has become a central topic in international transportation research due to growing concerns about climate change, energy consumption, and urban livability. Recent studies emphasize that sustainable transportation systems should not only reduce environmental impacts but also promote socially inclusive mobility opportunities and equitable access to urban services [10,11].

Nevertheless, to achieve sustainable transportation, it is essential to develop these three components and their sub-goals in an integrated manner [12,13]. The social dimension of sustainable transportation is achieved by integrating environmental and socio-human aspects into economic growth objectives, thereby targeting sustainable human development.

Recent literature also highlights that the social dimension of sustainable transportation includes issues such as accessibility, equity, public participation, and social inclusion in mobility systems [14,15].

Economic and environmental projects that fail to improve the social structure and ensure public participation cannot be successful or sustainable. Therefore, in order to achieve sustainable urban development through sustainable transportation, the social dimension must be considered a primary concern.

Sustainable transportation planning can only be realized by meeting individuals’ demand for urban accessibility through walking and cycling, and by promoting non-motorized modes of transport [6,16–21].

Active transportation modes such as cycling and walking have become key components of sustainable urban mobility strategies. Cycling contributes not only to environmental sustainability but also to improved public health, reduced congestion, and enhanced urban livability [11,15].

In this context, cycling, due to its environmental friendliness, lack of energy consumption, and absence of air and noise pollution, emerges as a driving force in achieving sustainable transportation [22,23]. However, despite investments in cycling infrastructure in many cities, low usage rates necessitate the evaluation of this mode of transport within the scope of social sustainability [24–27].

Recent studies indicate that infrastructure investments alone are often insufficient to increase cycling levels; social norms, safety perceptions, cultural attitudes, and behavioral motivations also play a significant role in shaping bicycle use [15,28]. For instance, Steg & Vlek (2009) demonstrated how cognitive and affective components jointly shape pro-environmental behaviour [29]. Similarly, Van Acker, Van Wee, and Witlox (2010) integrated social psychology with transport geography to explain variations in travel behaviour [30].

Building on these perspectives, contemporary sustainable mobility research increasingly integrates behavioral transportation theories with urban mobility studies to better understand how attitudes, values, and social norms influence transportation choices [10,31].

In this study, awareness is considered a multidimensional construct, encompassing not only knowledge but also attitudes and behavioral tendencies regarding sustainable transportation. This framing highlights the social dimension of mobility as more than a technical issue, but also as a cultural and psychological process.

Therefore, the aim of this study is to examine the effects of university students’ awareness levels, shaped by their fields of vocational education, on the development of the social dimension of sustainable transportation. The study also seeks to enhance students’ awareness regarding the social aspects of sustainable transportation and to promote the use of non-motorized modes of transport within the university context.

The social dimension of sustainable transportation aims to develop human-centered policies by reducing private vehicle usage and promoting public and non-motorized transportation [32–35]. While environmental sustainability focuses on ecological balance and economic sustainability on development, social sustainability is an indispensable component for the advancement of both [36].

Within this perspective, the social dimension of sustainable transportation can be understood through several key principles that promote socially inclusive mobility systems. These principles include equity, accessibility, health and safety, individual responsibility, integrated planning, and cultural values. Equity refers to providing transportation opportunities to all individuals without discrimination and ensuring affordability for different income groups [17,37,38]. Accessibility relates to individuals’ ability to reach desired destinations through efficient, affordable, and environmentally responsible transport systems [36,39–41]. Health and safety emphasize the benefits of active transportation modes such as walking and cycling for public health and traffic safety [8,42]. Individual responsibility highlights the role of personal awareness and lifestyle choices in reducing automobile dependency and promoting sustainable mobility practices [8,19,43–48]. Integrated planning refers to coordinating land-use and transportation systems in order to support sustainable mobility patterns, while cultural values and habits influence transportation preferences and the social acceptance of alternative mobility options [19,37,44,49,50]. These principles collectively constitute the conceptual framework of the social dimension of sustainable transportation examined in this study.

Sustainable transportation planning is contingent upon individuals’ self-awareness, recognition of their competencies, and the development of consciousness accordingly [51,52]. This awareness is shaped during family life, formal education, and particularly through vocational education processes [53]. Since vocational education is as influential as family upbringing in shaping transportation preferences, awareness regarding the social dimension of sustainable transportation is directly related to an individual’s educational background [54]. Especially at the university level, vocational training significantly contributes to the development of students’ awareness of the social dimension of sustainable transportation.

Universities are not only institutions of knowledge production but also bear the responsibility of cultivating environmentally conscious individuals who guide society [55,56]. The awareness university students gain regarding sustainable transportation represents a fundamental step toward societal development and sustainable urban growth [57]. Through the individuals they educate, universities have the potential to influence the social and cultural fabric of the communities they serve and to foster a society grounded in sustainability consciousness [58]. Hence, the adoption of sustainability principles by universities and their role as models for students play a critical role in raising societal awareness and promoting sustainable urban development [59–61].

Although the number of studies on sustainable transportation and cycling has increased considerably in recent years, relatively few studies have examined the social dimension of sustainable mobility, particularly in relation to university students’ awareness and attitudes toward bicycle use. Most existing studies primarily focus on environmental impacts, infrastructure provision, or travel behavior patterns, while the social and behavioral aspects of sustainable transportation remain comparatively underexplored. Understanding individuals’ awareness levels and how this awareness influences transportation behavior is therefore an important research area in the sustainable mobility literature.

In this study, awareness is defined as the extent to which individuals can recognize, interpret, and respond to the social and cultural implications of sustainable transportation. Awareness encompasses three main dimensions: cognitive (knowledge), affective (attitudes), and behavioral (practices) [29,31]. In this context, university students’ awareness levels are conceptualized as a multidimensional construct shaped by educational background, socio-cultural environment, and personal experiences.

Within this framework, the study aims to examine the awareness levels of university students regarding the social dimension of sustainable transportation. Specifically, the research investigates how awareness levels shaped by students’ vocational education fields contribute to the development of the social dimension of sustainable transportation and influence the promotion of bicycle use.

To achieve this aim, the study evaluates several indicators related to the social dimension of sustainable transportation, including equality, accessibility, health and safety, individual responsibility, integrated planning, and cultural values. By analyzing these indicators together with socio-demographic characteristics and educational backgrounds, the research seeks to provide a more comprehensive understanding of the social and behavioral factors influencing sustainable mobility awareness among university students.

The study also aims to increase awareness of the indicators related to the social dimension of sustainable transportation and to develop recommendations for promoting bicycle use in urban contexts. In this respect, the research intends to contribute to the development of planning and policy approaches that support sustainable mobility in university environments.

At Amasya University, a stratified sampling method was adopted to ensure that variations across vocational education fields were adequately represented, thereby strengthening the representativeness of the sample by capturing the heterogeneity among professional domains [38]. In addition, attitude analysis was employed, as this method allows for the measurement and quantification of perceptions and values influencing cycling behavior. Attitude analysis has been widely used in social psychology and transportation research to predict pro-environmental behaviors and to account for intergroup differences [62].

This study contributes to the sustainable transportation literature in several ways. First, it focuses specifically on the social dimension of sustainable transportation, an aspect that has received relatively limited attention compared to environmental and economic dimensions. Second, the study examines university students’ awareness levels within a campus-based context, providing empirical insights into how educational background and faculty differences influence perceptions of sustainable mobility. Third, by integrating social sustainability indicators with attitude analysis, the study offers a framework for understanding how awareness of social sustainability principles may shape students’ willingness to adopt cycling as a transportation alternative. These findings provide practical implications for sustainable mobility planning in university cities.

## 2. The purpose and methodology of the study

University students’ educational backgrounds and experiences directly shape their transportation preferences and attitudes toward sustainable mobility. Bicycle use, one of the fundamental components of sustainable transportation, is strongly influenced by social indicators such as equity, accessibility, integrated planning, individual responsibility, health and safety, as well as cultural values and habits [63]. In this context, the aim of the study is to identify students’ awareness levels based on their vocational education fields, to examine their consciousness regarding bicycle use, and to develop policy recommendations accordingly.

**Fig 1:**
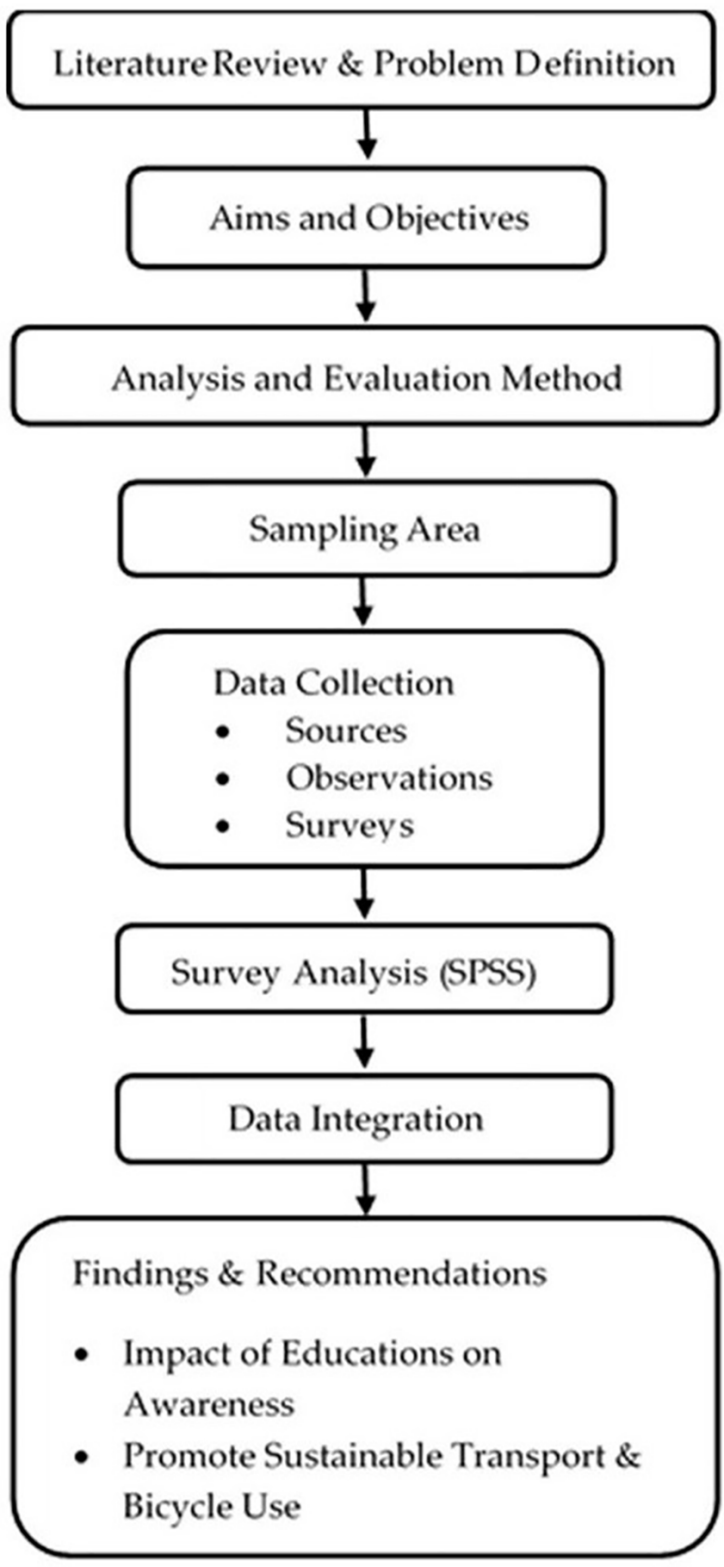
Study workflow.

In this context, the workflow followed in the study is presented in Fig 1.

In line with the stated purpose of the study, the main and supporting research questions that guide the study are presented below.

**Main research questions:**

- How are university students’ awareness levels regarding the social dimension of sustainable transportation defined and measured?
- How do vocational education processes in different faculties influence students’ awareness of the social dimension of sustainable transportation?
- How can students’ awareness levels be associated with their attitudes and preferences toward bicycle use?

**Fig 2:**
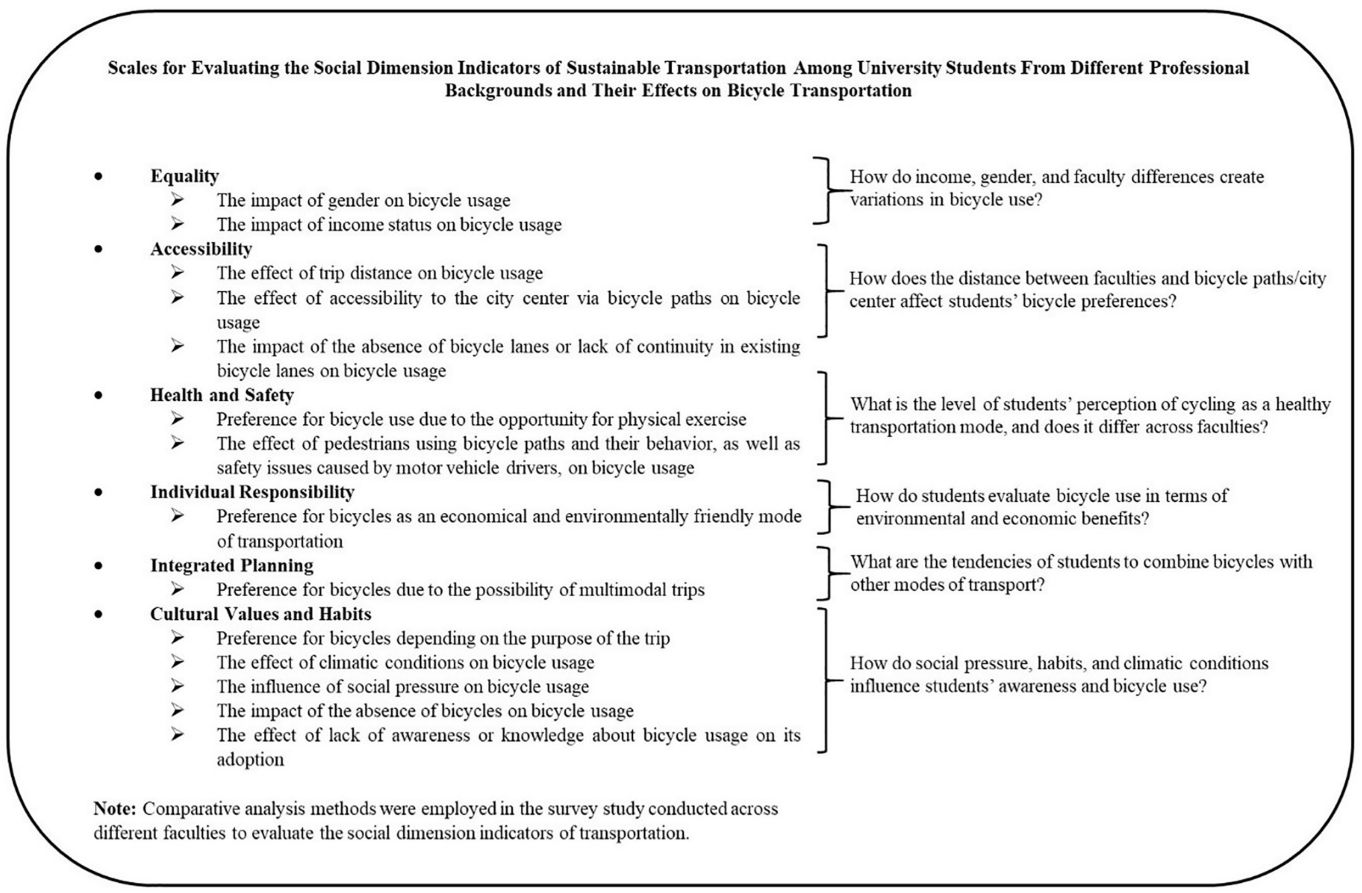
The subcomponents defined for addressing the research questions identified within the scope of the study.

To address the main research questions formulated within the scope of the study, sub-questions were developed based on the subcomponents of social sustainability. The components examined in the study constitute the foundation of the data collection, analysis, and interpretation processes, thereby enabling a systematic approach to the research questions. Each subcomponent is grounded in the theoretical framework established in the relevant literature and is presented in Fig 2. Fig 2 presents the conceptual framework of the study based on the social dimension of sustainable transportation. The framework illustrates the main components and subcomponents of social sustainability used in the analysis, including accessibility, equity, health, individual responsibility, cultural values, and societal pressure. These components provide the analytical basis for examining university students’ awareness of the social dimension of sustainable transportation and for structuring the data collection and interpretation processes.

Among these components, the societal pressure factor refers to the influence of broader social norms, cultural expectations, and collective attitudes on individuals’ transportation choices. In the context of sustainable mobility, this factor reflects how societal perceptions may encourage or discourage the adoption of transportation modes such as bicycle use. Therefore, societal pressure in this study does not directly refer to income level; rather, it represents the role of shared social values and community perceptions in shaping mobility behaviors.

In this study, awareness is conceptualized as a multidimensional construct encompassing cognitive (knowledge), affective (attitude), and behavioral (practice) dimensions. Six indicators representing the social dimension of sustainable transportation were used for measurement. These indicators were evaluated through a combination of Likert-type survey questions, field observations, and comparative statistical analyses conducted using SPSS. Each indicator was measured through multiple survey questions using a three-point Likert scale ranging from “strongly disagree” to “strongly agree,” where higher scores indicate higher levels of awareness regarding the social dimension of sustainable transportation. This mixed approach enabled the integration of qualitative and quantitative data, allowing students’ perceptions to be associated with statistically significant results (p < 0.05).

### 2.1. Measurement tool and ındicators

The questionnaire used in this study was developed based on the literature on the social dimension of sustainable transportation. The survey items were derived from previous studies addressing sustainable mobility, transportation behavior, and social sustainability indicators.

The indicators used in this study were derived from the theoretical framework of socially sustainable transportation discussed in the literature.

The measurement tool consisted of items grouped under six indicators representing the social dimension of sustainable transportation: equality, accessibility, health and safety, individual responsibility, integrated planning, and cultural values and habits.

In total, 26 questions were included in the questionnaire administered in this study. Five of these questions were related to demographic characteristics, while 21 questions were designed to measure the social dimension of sustainable transportation.

These 21 items were grouped under six indicators representing the social dimension of sustainable transportation: equality, accessibility, health and safety, individual responsibility, integrated planning, and cultural values and habits. The distribution of the items according to these indicators is presented in Table 1.

**Table 1:** Indicators and number of ıtems used in the survey.

Indicator (Social Dimension of Sustainable Transportation)	Number of Items
Equality	3
Accessibility	5
Health and Safety	3
Individual Responsibility	2
Integrated Planning	6
Cultural Values and Habits	2
**Total**	**21**

**Fig 3:**
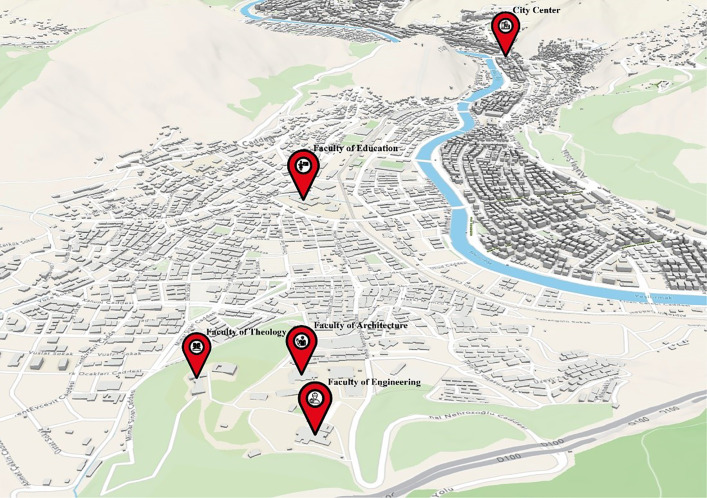
Locations of Faculties within the city (Author’s own elaboration based on OpenStreetMap spatial data using ArcGIS Pro 3.4.0 and Adobe Illustrator 2026).

The selection of faculties included in the study was based on both representativeness and spatial distribution considerations. The number of students enrolled in the Faculty of Education of Amasya University (1845 students) is approximately equivalent to the total number of students studying in the Faculties of Architecture (409 students), Engineering (411 students), and Theology (412 students). This numerical balance made it possible to achieve a relatively homogeneous distribution among students participating in the survey. In addition, the Faculty of Education is located close to the city center and outside the main campus of Amasya University. This spatial distribution enabled the study to capture the awareness levels of students studying in faculties located in different parts of the city, thereby providing a broader perspective on sustainable transportation awareness (Fig 3).

### 2.2. Sampling and data collection

The total target population was determined to be 3,077. Based on a 95% confidence level and a 5% margin of error, the sample size was calculated as 341 [51,64].


$$\text{n}=\left(\text{N}*{\text{Z}}^{\text{2}}*\text{p}*(\text{1}-\text{p})\right)/\left((\text{N}-\text{1})*{\text{e}}^{\text{2}}+{\text{Z}}^{\text{2}}*\text{p}*(\text{1}-\text{p})\right)$$

- n = Required sample size
- N **=** Population size (N = 3,077)
- Z = Z-score corresponding to the desired confidence level (e.g., 1.96 for 95%)
- p = Estimated proportion of the population (assumed as 0.5)
- e = Margin of error (e.g., 0.05 for 5%)

$$𝐧=\left(3,077*1.96^{2}*0.5*\left(1-0.5\right)\right)/\left(\left(3,077-1\right)*0.05^{2}+1.96^{2}*0.5*\left(1-0.5\right)\right)=341\approx 300$$

However, in practice, a total of 300 students were surveyed to ensure a more homogeneous distribution across strata. The questionnaires were evenly distributed between students who use bicycles and those who do not, and were proportionally allocated among the faculties based on their student numbers (Table 2).

**Table 2:** Number of questionnaires distributed in proportion to stratum sizes.

Layer	Number of Subjects	Layer Weight	Stratum Sample Size
	N_k_	W_k_ = N_k_/N	n_k_ = W_k_ × N
Faculty of Education	1,845	W_1_ = 1,845/3,077	n_1_ = 0.5996 × 300 = 180
Faculty of Engineering	411	W_2_ = 411/3,077	n_2_ = 0.1336 × 300 = 40
Faculty of Architecture	409	W_3_ = 409/3,077	n_3_ = 0.1329 × 300 = 40
Faculty of Theology	412	W_4_ = 412/3,077	N_4_ = 0.1339 × 300 = 40
**Total**	N = 3077	1	300

Participants were randomly approached in different areas of the university campus, and students who voluntarily agreed to participate were included in the study. The survey was conducted during the active academic period between April 7 and May 2, 2025. Data were collected through face-to-face questionnaires administered to participants aged 18 years and older. The questionnaires were completed individually by the respondents under the supervision of the researchers to ensure that the questions were clearly understood. Participation in the study was voluntary and anonymous, and no personally identifiable information was collected.

Ethical approval for the study was obtained from the relevant ethics committee (Ondokuz Mayıs University, Social and Human Sciences Research Ethics Committee, Approval No: 2025−300) prior to data collection. All participants were informed about the purpose of the study, the voluntary nature of participation, and the confidentiality of their responses. Informed consent was obtained verbally from all participants before the questionnaires were administered, as the survey was conducted face-to-face in public areas of the university campus. Only individuals aged 18 years and older were included in the study; therefore, no parental or guardian consent was required.

Because of time, cost, and labor constraints, it is not feasible to study the entire student population to examine the level of awareness regarding sustainable transportation. For this reason, it was deemed appropriate to select a sample to represent the student population. First, the sample size was determined, and then, in order to assess the level of awareness of university students in the sample area regarding sustainable transportation, a grouped stratified sampling method, which is important for representing the general structure of university students, was used.

**Fig 4:**
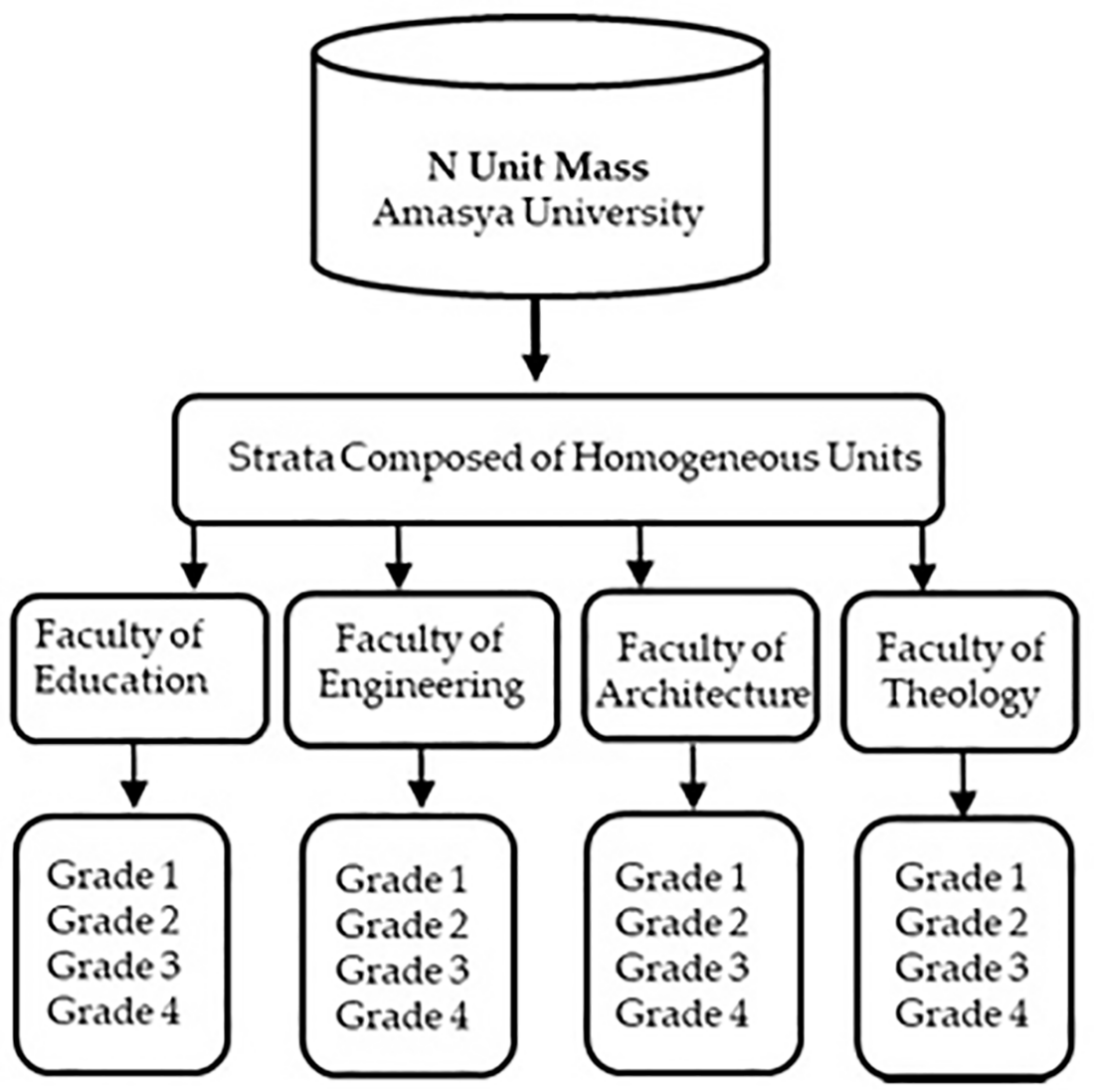
Population, strata, and homogeneous groups selected in the sample area.

Fig 4 illustrates the sampling method, in which each unit of the population belongs to only one stratum and is divided into subgroups such that the intra-stratum variation is minimized and the inter-stratum variation is maximized so that no population unit is excluded. The sample was drawn from each stratum separately and independently of each other; this is called stratified sampling [65].

### 2.3. Reliability and validity analysis

Reliability analysis was conducted to evaluate the internal consistency of the measurement tool. In this context, Cronbach’s Alpha coefficient was calculated using SPSS 22.0. Exploratory factor analysis was also performed to examine the construct validity of the measurement indicators. The Kaiser–Meyer–Olkin (KMO) measure of sampling adequacy and Bartlett’s Test of Sphericity were used to determine whether the dataset was suitable for factor analysis.

The reliability and validity analyses were conducted separately for each indicator representing the social dimension of sustainable transportation. The results of these analyses are presented in the Survey Findings section.

It should be noted that the primary objective of this study was not to develop a new measurement scale but to evaluate university students’ awareness levels regarding the social dimension of sustainable transportation through theoretically defined indicators.

In this study, the attitude analysis method was employed to understand, predict, and interpret individuals’ attitudes and behaviors. Measuring attitudes is often challenging, as individuals do not always provide explicit or accurate responses regarding their behaviors and attitudes. Therefore, attitude analysis is significant for quantifying social structure data that cannot be directly measured numerically [66]. Since bicycle trips are based on user preferences, the data are inherently relative in nature. For this reason, the study adopted a mixed-methods approach, combining both qualitative and quantitative techniques. The criteria used to assess university students’ awareness levels of sustainable transportation and their relationship with qualitative and quantitative data are summarized in Table 3.

**Table 3:** Measurement criteria for examining university students’ awareness levels regarding sustainable transportation.

Indicator	Qualitative	Quantitative	Measurement Techniques
	(Measurable—Spatial)	(Non-Measurable—Non-Spatial)	
**Equality**		*	Survey on gender, income, education, and trip distance effects on bicycle use.
**Accessibility**	*	*	Digital maps for accessibility; survey on presence, sufficiency, and continuity of bicycle paths.
**Health and Safety**	*	*	Field observations; survey on transport awareness, driver/pedestrian attitudes, traffic compliance, social supervision, and public safety.
**Individual Responsibility**		*	Survey on environmental, economic, and short-distance benefits of cycling and personal responsibility.
**Integrated Planning**	*		Field observations and maps to assess path integration; survey on parking and infrastructure sufficiency.
**Cultural Values and Habits**		*	Survey on cycling duration, seasonal effects, bicycle choice purpose, preferred modes, car ownership effect, and societal attitudes.

### 2.4. Statistical analysis

The data were analyzed using SPSS 22.0 through frequency analysis and chi-square tests. Only relationships that were statistically significant at the p < 0.05 level were reported [67]. In the comparative analyses, Pearson’s Chi-square test was applied to examine whether statistically significant relationships existed between categorical variables. In this test, expected frequencies were automatically calculated based on the distribution of observed values within contingency tables according to the standard chi-square procedure. Using this method, relationships between variables such as gender, income level, and faculty location and the awareness indicators were identified. In this study, the variable of “income” was not limited to students’ personal earnings, as the majority of university students do not have independent income. Instead, household income levels reported by the students, based on their family’s monthly earnings, were considered. This approach allows the variable to more accurately reflect students’ socio-economic backgrounds and their potential influence on mobility preferences [9,12].

The analyses revealed significant relationships between gender and perceptions of health and safety, income level and accessibility awareness, and cultural awareness and the equity indicator. These findings indicate that the indicators representing the social dimension of sustainable transportation are interrelated rather than independent. The study therefore aims to reveal how university students’ awareness levels, shaped by their fields of study, influence the social dimension of sustainable transportation. Particular attention is given to bicycle transportation as a sustainable, accessible, and environmentally friendly mobility alternative within university environments. The findings provide insights that may support the development of policies and practical initiatives at universities aimed at promoting bicycle use and encouraging sustainable transportation practices.

## 3. Research findings

In this study, the city of Amasya was selected as the sample area. The topographical features of Amasya have defined and limited the city’s expansion over the centuries. Amasya province is located in the south of the Canik Mountains, in the central part of Northern Anatolia, between (34°57′06″–36°31′53″) Eastern Longitude and (41°04′54″–40°16′16″) Northern Latitude (URL 1) [68].

**Fig 5:**
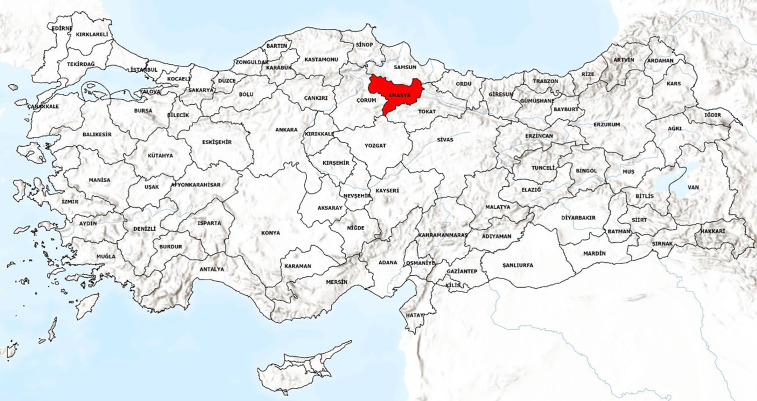
Map of Amasya province (Author’s own elaboration based on OpenStreetMap spatial data using ArcGIS Pro 3.4.0 and Adobe Illustrator 2026).

Within this region, Amasya province is located west of the confluence of the Yeşilırmak/Iris and the Tersakan river that irrigates the Merzifon plain. Amasya is a city on the Black Sea border and is surrounded by Samsun, Tokat, Çorum, and Yozgat (Fig 5).

According to 2024 data, Amasya is a small-sized city with a population of 342,378, where university students play an important role in shaping the city’s **social structure, economic** dynamics, and spatial development (URL 2) [69].

**Fig 6:**
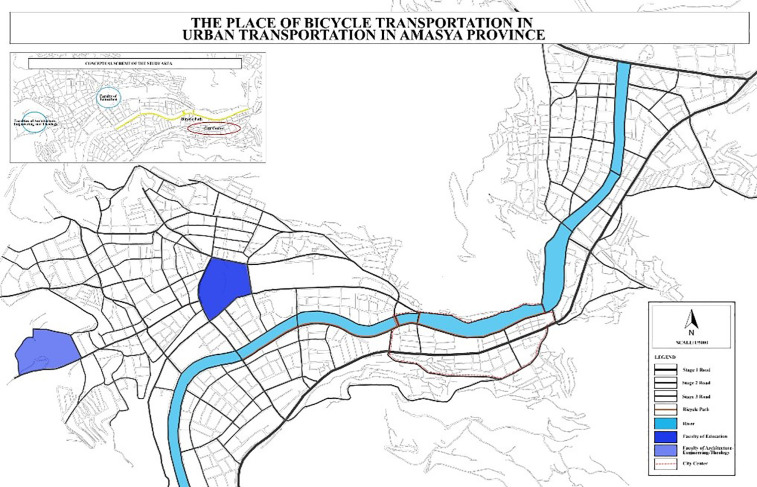
Bicycle paths in Amasya (Source: Author’s own elaboration based on OpenStreetMap spatial data using ArcGIS Pro 3.4.0 and Adobe Illustrator 2026).

Fig 6 shows the spatial distribution of bicycle use in the city of Amasya. Bicycle activity is mainly concentrated along the Yeşilırmak riverside corridor. In contrast, bicycle use remains limited in other parts of the city. This spatial pattern indicates that bicycle transportation has not yet become a widely integrated mobility option within the broader urban transportation system.

### 3.1. Observations in the field

Field observations indicate that bicycle paths in Amasya are mainly located along the Yeşilırmak riverside corridor (Fig 6). However, these bicycle routes remain spatially limited and are not integrated into the broader urban transportation network. As a result, bicycle use does not extend across the entire city.

University students generally travel between the university campus, student housing areas, and the city center, which form the main daily mobility routes. Despite this mobility pattern, the bicycle paths located along the riverside do not establish a direct spatial connection with major university facilities such as the Faculties of Education, Architecture, Engineering, and Theology. This lack of connectivity indicates that the existing bicycle infrastructure does not adequately support students’ daily mobility needs.

**Fig 7:**
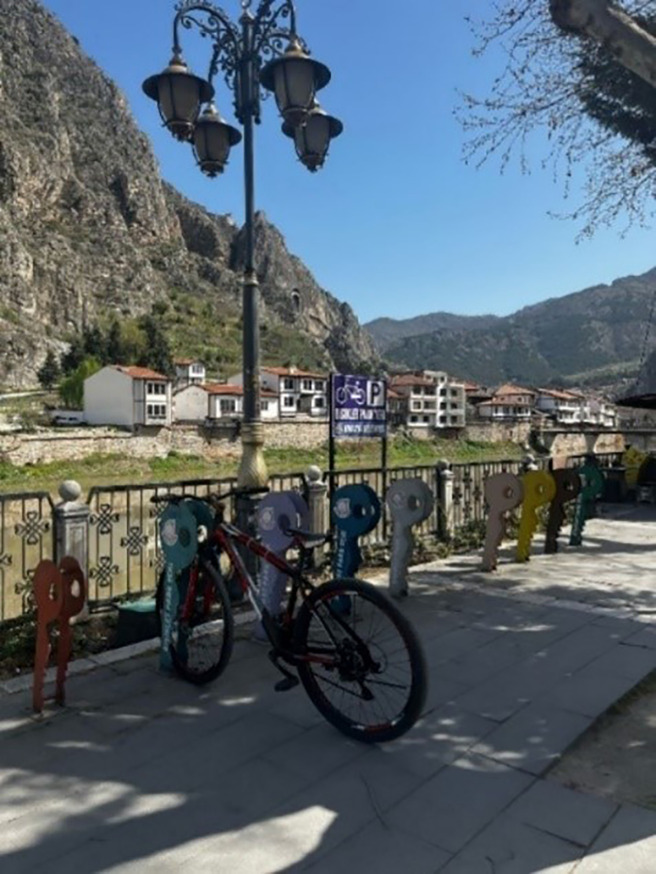
Urban bicycle parking spaces. (Source: Photograph taken by the author (2025)).

**Fig 8:**
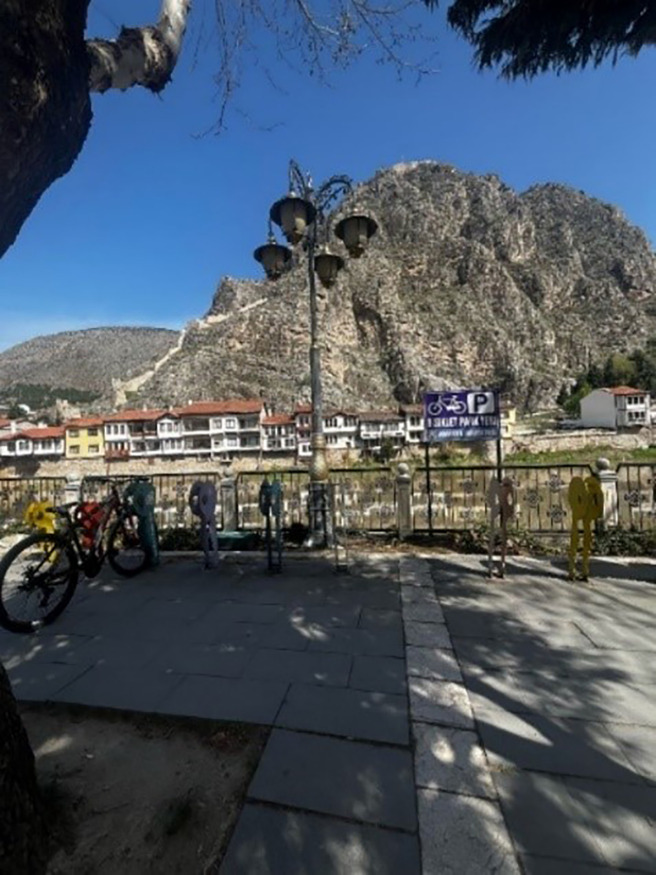
Urban bicycle parking spaces. (Source: Photograph taken by the author (2025)).

As a result of the observations, it was determined that bicycle parking spaces are only available in a certain areas and are insufficient throughout the city (Figs 7 and 8) The limited provision of bicycle parking facilities across the city appears to negatively affect the daily commuting patterns of cyclists, while also diminishing the effectiveness of transportation policies intended to encourage bicycle use.

**Fig 9:**
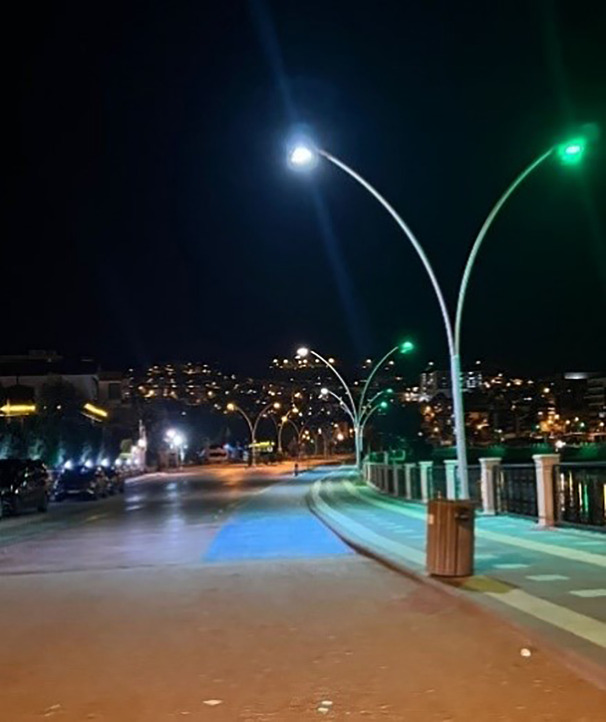
Discontinuous bicycle paths. (Source: Photograph taken by the author (2025)).

**Fig 10:**
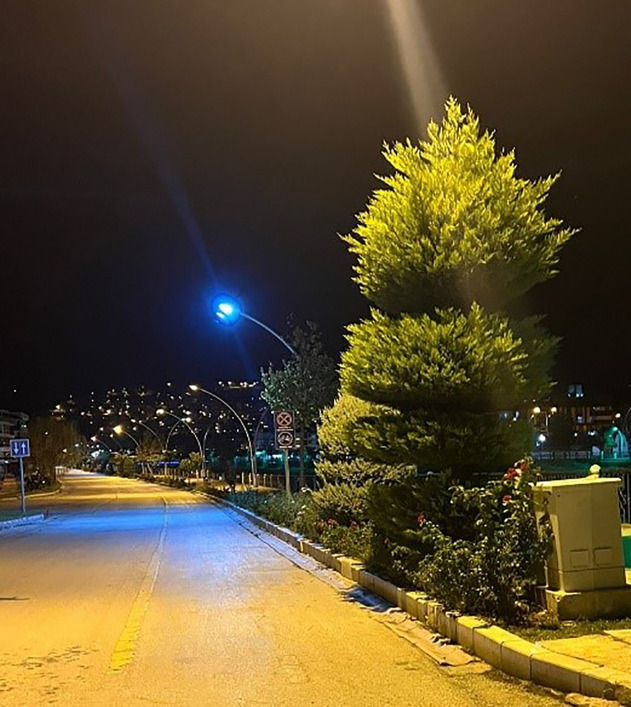
Bicycle paths are not distinguished from the roadway by factors such as color, pavement, etc. (Source: Photograph taken by the author (2025)).

In addition, the existing bicycle paths do not provide pedestrian-bicycle interconnectivity due to a lack of continuity (Fig 9), and many bicycle paths lack color, pavement, and other features that distinguish them from vehicular roads (Fig 10). These deficiencies not only compromise the safety of both cyclists and pedestrians but also hinder the effective implementation of sustainable transportation policies aimed at promoting pedestrian-cyclist integration within the urban context.

### 3.2. Survey findings

This section presents the findings obtained from the survey conducted with university students in order to examine their awareness levels regarding the social dimension of sustainable transportation. The analysis focuses on indicators related to equality, accessibility, health and safety, individual responsibility, integrated planning, and cultural values and habits. Reliability and validity analyses were first conducted to evaluate the internal consistency and structural suitability of the indicators. Subsequently, comparative analyses were performed to examine whether significant differences existed between students who use bicycles and those who do not. The findings obtained from these analyses are presented in the following subsections.

#### 3.2.1. Reliability and validity results.

Reliability analysis was conducted to evaluate the internal consistency of the measurement tool. In this context, Cronbach’s Alpha coefficient was calculated using SPSS 22.0. Exploratory factor analysis was also performed to examine the construct validity of the measurement indicators. The Kaiser–Meyer–Olkin (KMO) measure of sampling adequacy and Bartlett’s Test of Sphericity were used to determine whether the dataset was suitable for factor analysis. The reliability and validity analyses were conducted separately for each indicator representing the social dimension of sustainable transportation.

The results of these analyses are presented in the Results section. It should be noted that the primary objective of this study was not to develop a new measurement scale but to evaluate university students’ awareness levels regarding the social dimension of sustainable transportation through theoretically defined indicators.

Reliability and validity analyses were conducted for each indicator representing the social dimension of sustainable transportation. Cronbach’s Alpha coefficients ranged between 0.500 and 0.921, indicating acceptable to high internal consistency for most indicators. The Kaiser–Meyer–Olkin (KMO) values ranged between 0.230 and 0.806. Bartlett’s Test of Sphericity was statistically significant for all indicators (p < 0.05), indicating that the dataset was suitable for factor analysis. The results of the reliability and validity analyses are presented in Table 4.

**Table 4:** Reliability and validity results of the indicators.

Indicator	Cronbach Alpha	KMO	Bartlet Sig.
**Equality**	0.725	0.500	0.000
**Accessibility**	0.921	0.806	0.006
**Health and Safety**	0.568	0.411	0.003
**Individual Responsibility**	0.911	0.500	0.000
**Integrated Planning**	0.917	0.641	0.000
**Cultural Values and Habits**	0.500	0.230	0.001

These results indicate that the indicators used in the study provide an acceptable level of reliability for examining the social dimension of sustainable transportation.

#### 3.2.2. Descriptive and comparative findings.

The main purpose of this part of the study is to reveal the effects of the social dimension principles of sustainable transportation on cycling. In this context, the factors in the social dimension that affect the preferences of the surveyed individuals for cycling for urban transportation were observed, as well as their degree of influence.

Regarding the general demographics of the respondents, 63.4% of the respondents were women, and 36.6% were men (Table 5).

**Table 5:** Genders of the respondents.

What is Your Gender?		
Woman	Men	Total
Percent	Percent	Percent
63.4%	36.6%	100%

Of the surveyed students, 91.6% did not own a private vehicle (Table 6).

**Table 6:** Private vehicle ownership of the respondents.

Do You Have a Private Car?		
Yes	No	Total
Percent	Percent	Percent
8.4%	91.6%	100%

In the survey, Turkish Statistical Institute (TurkStat) data were taken into consideration for income grouping. Among the students participating in the survey, 33.4% are in the medium-income group, 28.3% are in the medium-low-income group (TRY 17.000-25.000), and 33.4% are in the medium-high-income group (TRY 33.001–41.000). The percentage of students with low income is 6.6%, and the percentage of students with high income is 8.3% (Table 7).

**Table 7:** Analysis of income status of the respondents.

What is Your Income?					
TRY Less than 17.000 (Low)	TRY 17.001–25.000 (Medium-Low)	TRY 25.001–33.000 (Medium)	TRY 33.001–41.000 (Medium-High)	TRY 41.001+ (High)	Total
Percent	Percent	Percent	Percent	Percent	Percent
6.6%	28.3%	33.4%	23.40%	8.3%	100%

- **Evaluation of the social dimension of sustainable transportation according to the equality parameter**

The comparative analysis indicated that gender and cycling status were related (p = 0.001). As seen in Table 7, 40.0% of the bicycle users are male and 60.0% are female (Table 8).

**Table 8:** Effects of gender on cycling.

Values	Do You Use a Bicycle?			
	Yes	No	Total	*p*
	Percent	Percent	Percent	
**Woman**	60.0%	68.7%	63.4%	**0.001**
**Men**	40.0%	31.3%	36.6%	
**Total**	100.0%	100.0%	100.0%	

Income status and bicycle use were also significantly related (p = 0.000). The findings show that 6.6% of bicycle users had an income of less than minimum wage, 28.3% had an income of TRY 17.000–25.000, 33.4% had an income of TRY 25.000–33.000, 23.4% had an income of TRY 33.001–41.000, and 8.3% had an income of TRY 41.000 and above (Table 9).

**Table 9:** The effect of income status on bicycle use.

Values	Do You Use a Bicycle?			
	Yes	No	Total	*p*
	Percent	Percent	Percent	
Less than 17.000	8.6%	4.6%	6.6%	**0.000**
17.001.–25.000	34.0%	22.6%	28.3%	
25.001–33.000	30.6%	36.0%	33.4%	
33.001–41.000	22.6%	24.0%	23.4%	
41.001+	7.3%	9.3%	8.3%	
**Total**	**100.0%**	**100.0%**	**100.0%**

- **Evaluation of the social dimension of sustainable transportation according to the accessibility parameter**

The distance of bicycle use was significantly related to the faculty of study (p = 0.001). The results show that 47,3% of the students who use bicycles have a travel distance of less than 3 km, 34.0% have a travel distance of 3.01–6 km, 11.3% have a travel distance of 6.01–9 km, 6% have a travel distance of 9.01–12 km, and 0.0% have a travel distance of more than 12 km. Since cycling relies on physical strength, it is less preferred for long distances (Table 10).

**Table 10:** Distance traveled by bicycle according to faculty of study.

What Is the Distance of Your Cycling Journey?							
Faculties	Less than 3 km	3.01–6 km	6.01–9 km	9.01–12 km	12.01 km+	Total	*p*
	%	%	%	%	%	%	
Education	51.1%	35.5%	7.7%	5.5%	0.0%	100.0%	**0.001**
Architecture	40.0%	32.0%	20.0%	8.0%	0.0%	100.0%	
Engineering	45.0%	30.0%	15.0%	10%	0.0%	100.0%	
Theology	33.3%	33.3%	26.6%	0%	0.0%	100.0%	
Total	47.3%	34.0%	11.3%	6.0%	0.0%	100.0%	

In the comparative analysis of the problems students encountered related to accessibility during bicycle trips, the results show that 65% of the bicycle users in the Faculties of Architecture and Engineering agree that there is a lack of bicycle paths accessing the center. This rate is 50% among students in the Faculty of Education and 53.3% among those in the Faculty of Theology (Table 11). However, accessibility problems encountered during bicycle journeys (e.g., lack of bicycle paths, lack of continuity of existing roads, distance to destination) were not significantly related to the faculty of study (p > 0.05), so this relationship was not evaluated further.

**Table 11:** Problems related to accessibility in bicycle use according to faculty of study.

What Is Your Experience with Problems Related to Access While Cycling?						
Faculties		I agree	Undecided	Disagree	Total	*p*
		%	%	%	%	
Lack of a bicycle path accessing the center	Education	50.0%	17.7%	32.2%	100.0%	
	Architecture	64.0%	12.0%	24.0%	100.0%	
	Engineering	65.0%	15%	20%	100.0%	**0.011**
	Theology	53.3%	13.3%	33.3%	100.0%	
	Total	54.6%	16.0%	29.3%	100.0%	

In the comparative analysis of the proportion of students who do not use bicycles due to accessibility problems, 51.1%, 53.3%, 60.0%, and 28.0% of students studying in the Faculties of Education, Architecture, Engineering, and Theology, respectively, reported not using bicycles due to long travel distances (Table 12).

**Table 12:** Comparative analysis of agreement with accessibility-related reasons for not using bicycles according to the faculty of study.

What Are the Access-Related Reasons for not Cycling?						
Values		I agree	Undecided	Disagree	Total	*p*
		%	%	%	%	
Since my destinations are far away	Education	51.1%	18.9%	30.0%	100.0%	0.000
	Architecture	53.3%	20.0%	26.6%	100.0%	
	Engineering	60.0%	15.0%	25.0%	100.0%	
	Theology	28.0%	8.0%	64.0%	100.0%	
	**Total**	**48.7**%	**16.7**%	**34.6**%	**100.0**%	
Lack of bicycle paths, paths and continuity of existing roads	Education	36.7%	30.0%	33.3%	100.0%	0.000
	Architecture	66.6%	13.3%	20.0%	100.0%	
	Engineering	30.0%	25.0%	45.0%	100.0%	
	Theology	20.0%	32.0%	48.0%	100.0%	
	**Total**	**36.0**%	**28.0**%	**36.0**%	**100.0**%	
Lack of a bicycle path accessing the city center	Education	38.9%	30.0%	31.1%	100.0%	0.001
	Architecture	60.0%	20.0%	20.0%	100.0%	
	Engineering	35.0%	35.0%	30.0%	100.0%	
	Theology	20.0%	20%	60.0%	100.0%	
	**Total**	**37.3**%	**28.0**%	**34.7**%	**100.0**%	

Furthermore, 36.7%, 66.6%, 30.0%, and 20% of students from the same faculties reported not using bicycles due to the lack of bicycle paths, and the lack of continuity of existing roads (Table 12).

Finally, 38.9%, 60%, 35.0%, and 20% of the respondents from the Faculties of Education, Architecture, Engineering, and Theology, respectively, reported not using bicycles due to the lack of a bicycle path providing access to the center (Table 12).

- **Evaluation of the social dimension of sustainable transportation according to the health and safety parameter**

In evaluating the effects of the health and safety parameter of the social dimension of sustainable transportation on bicycle use, 77.4% of respondents stated that they prefer bicycle transportation because it provides an opportunity to exercise (Table 13). Therefore, it is inferred that the level of awareness of bicycle users regarding personal health is high.

**Table 13:** Level of agreement with health-related reasons for preferring cycling.

What Are Your Views on the Health and Safety Aspects of Cycling?				
Values	I Agree	Undecided	Disagree	Total
	%	%	%	%
I use a bicycle because I can do sports	77.4%	12.0%	10.6%	100.0%

In the comparative analysis conducted to determine the proportion of bicycle users who encountered health and safety problems, a significant relationship was found and taken into consideration (p = 0.000). Specifically, 37.7%, 72.0%, 65.0%, and 53.4% of those studying at the Faculties of Education, Architecture, Engineering, and Theology, respectively, have experienced problems with pedestrians using the bicycle path (Table 14).

**Table 14:** Health and safety problems encountered while cycling according to the faculty of study.

What Are the Health and Safety Related Problems You Experience While Cycling?						
Values		I Agree	Undecided	Disagree	Total	*p*
		%	%	%	%	
Pedestrians using bicycle paths	Education	37.7%	27.7%	34.4%	100.0%	0.000
	Architecture	72.0%	24.0%	4.0%	100.0%	
	Engineering	65.0%	20.0%	15.0%	100.0%	
	Theology	53.4%	40.0%	6.6%	100.0%	
	**Total**	**48.6**%	**27.3**%	**24.0**%	**100.0**%	
Negative behavior of pedestrians towards cyclists	Education	45.5%	32.2%	22.2%	100.0%	0.000
	Architecture	72.0%	16.0%	12%	100.0%	
	Engineering	55.0%	25.0%	20.0%	100.0%	
	Theology	60.0%	20.0%	20.0%	100.0%	
	**Total**	**52.6**%	**27.3**%	**20.0**%	**100.0**%	
Negative behavior of vehicle drivers	Education	38.8%	32.2%	28.8%	100.0%	0.000
	Architecture	64.0%	24%	12%	100.0%	
	Engineering	75.0%	15.0%	10.0%	100.0%	
	Theology	53.4%	33.3%	13.3%	100.0%	
	**Total**	**49.3**%	**28.7**%	**30.0**%	**100.0**%	

A significant relationship was also found between the faculty of study of students who use bicycles and the problems they experience (p = 0.000): 45.5%, 72.0%, 55.0%, and 60.0% of those studying at the Faculties of Education, Architecture, Engineering, and Theology reported problems due to the negative attitudes of pedestrians toward cyclists (Table 14).

In the comparative analysis of the effects of the negative behaviors of vehicle drivers on bicycle use, a significant relationship was found (p = 0.000), where 38.8%, 64.0%, 75.0%, and 53.4% of students studying at the Faculties of Education, Architecture, Engineering, and Theology had experienced such problems (Table 14).

- **Evaluation of the social dimension of sustainable transportation according to the individual responsibility parameter**

In the comparative analysis conducted to determine the effects of individual responsibility and awareness level on bicycle use, a significant relationship emerged (p = 0.002). The results of the analysis show that 52.6% of the respondents use bicycles because it is economical, 46.0% because it is an environmentally friendly means of transportation, and 36.0% because the travel distance is short (Table 15).

**Table 15:** Analysis of individual responsibility influencing the preference for cycling.

What Are Your Views on Individual Responsibility in Choosing to Use Bicycles?					
Values	I agree	Undecided	Disagree	Total	*p*
	%	%	%	%	
Because it is economical	52.6%	16.6%	30.6%	100.0%	0.002
Because it is environmentally friendly	46.0%	20.6%	33.3%	100.0%	
Since my journey was short	36.0%	15.0%	52.0%	100.0%	

A preference for bicycles due to their economic value was significantly related to the faculty of study (p = 0.000). The results show that 50.0%, 60.0%, 70.0%, and 33.3% of those studying at the Faculties of Education, Architecture, Engineering, and Theology, respectively, prefer cycling because it is economical (Table 16).

**Table 16:** Analysis of the preference for cycling according to the faculty of study.

What Are Your Views on Individual Responsibility in Choosing to Use Bicycles?						
Faculties		I Agree	Undecided	Disagree	Total	*p*
		%	%	%	%	
Because it is economical	Education	50.0%	11.1%	38.9%	100.0%	**0.000**
	Architecture	60.0%	20.0%	20.0%	100.0%	
	Engineering	70.0%	10.0%	20.0%	100.0%	
	Theology	33.3%	33.3%	33.3%	100.0%	
	**Total**	**52.6**%	**16.6**%	**30.8**%	**100.0**%	
Because it is environmentally friendly	Education	61.1%	18.9%	20.0%	100.0%	**0.001**
	Architecture	80.0%	20.0%	0.0%	100.0%	
	Engineering	50.0%	30.0%	20.0%	100.0%	
	Theology	46.7%	20.0%	13.3%	100.0%	
	**Total**	**46.0**%	**20.7**%	**33.3**%	**100.0**%	
Since my journey was short	Education	60.0%	16.6%	23.4%	100.0%	**0.002**
	Architecture	60.0%	20.0%	20.0%	100.0%	
	Engineering	45.0%	15.0%	40.0%	100.0%	
	Theology	40.0%	20.0%	40.0%	100.0%	
	**Total**	**36.0**%	**12.0**%	**52.0**%	**100.0**%	

Preferring cycling because it is an environmentally friendly mode of transportation was significantly related to the faculty of study (p = 0.001). The results show that 61.1%, 80.0%, 50.0%, and 46.7% of those studying at the Faculties of Education, Architecture, Engineering, and Theology, respectively, prefer cycling because it is environmentally friendly (Table 16).

A short travel time was the reason for preferring cycling in 60.0%, 60.0%, 45.0%, and 40.0% of those studying at the Faculties of Education, Architecture, Engineering, and Theology, respectively (p = 0.002, Table 16).

- **Evaluation of the social dimension of sustainable transportation according to the integrated planning parameter**

In the evaluation of the integration between transportation modes in line with integrated planning principles, only 17.3% of the respondents who use bicycles prefer them for connecting trips (Table 17).

**Table 17:** Transit status of bicycle users during their journeys.

Do You Transfer When Cycling?		
Yes	No	Total
**%**	**%**	**%**
17.3%	82.7%	100.0%

In the comparative analysis conducted to determine the type of transportation preferred by respondents who transfer while using bicycles, a significant relationship was found (p = 0.001). The results of the analysis show that 69.3% of the respondents prefer the bus/minibus, 30.7% prefer automobiles, and 50.0% prefer other modes of transportation (Table 18).

**Table 18:** Type of transportation preferred by bicycle users for connecting trips.

When You Cycle, How Often Do You Use Each Mode of Transportation for Your Connecting Journeys?					
Values	Frequently	Sometimes	Never	Total	*p*
	%	%	%	%	
Bus/Minibus	69.3%	15.3%	15.3%	100.0%	**0.001**
Special Vehicle	30.7%	38.4%	34.6%	100.0%	
Other	50.0%	30.7%	19.2%	100.0%	

Students studying at the Faculty of Architecture account for the highest proportion of those who transfer, with 28.0%. Students in the Faculty of Education do not need to because their faculty is the closest to the city center. In the Faculty of Theology, this rate is 0.0% (Table 19).

**Table 19:** Analysis of transferring while cycling according to the faculty of study.

Do You Transfer When Cycling?				
Faculties	Yes	No	Total	*p*
	%	%	%	
Education	17.7%	82.2%	100.0%	**0.000**
Architecture	28.0%	72.0%	100.0%	
Engineering	15.0%	85.0%	100.0%	
Theology	0.0%	100.0%	100.0%	
**Total**	17.3%	82.6%	100.0%

- **Evaluation of the social dimension of sustainable transportation according to the cultural values and habits parameter**

In the evaluation of the effects of cultural values and habits on bicycle use, 30.0% of bicycle users have been using bicycles for 0–5 years, 26.6% for 6–10 years, 23.3% for 11–15 years, 15.3% for 16–20 years, and 4.6% for 21 years or more (Table 20). Given that the majority of university students fall within the 18–24 age group, the duration of bicycle use largely reflects cycling habits developed during childhood and adolescence. This finding suggests that early experiences with cycling play a formative role in shaping current awareness levels regarding the social dimension of sustainable transportation.

**Table 20:** Duration of bicycle use table.

How Long Have You Been Cycling?						
Values	0–5 Years	6–10 Years	11–15 Years	16–20 Years	21 Years and Above	Total
	%	%	%	%	%	%
30.0%	26.6%	23.3%	15.3%	4.6%	100%

The results of the comparative analysis of respondents who use bicycles and the faculty of study indicate that 72.2%, 68.0%, 50.0%, and 26.6% of the students studying at the Faculties of Education, Architecture, Engineering, and Theology, respectively, frequently use bicycles to engage in socio-cultural activities (Table 21). Since p > 0.05 in the comparison of bicycle use to go to the workplace, shopping, and school/education facility with the faculty of study, the relationship was not significant and was not evaluated further.

**Table 21:** Stated bicycle trip purposes according to the faculty of study.

For What Purpose Do You Use Bicycles?						
Faculties		Frequently	Sometimes	Never	Total	*p*
		%	%	%	%	
Socio-cultural	Education	72.2%	16.7%	11.1%	100.0%	**0.000**
	Architecture	68.0%	20.0%	20.0%	100.0%	
	Engineering	50.0%	25.0%	25.0%	100.0%	
	Theology	26.6%	33.4%	40.0%	100.0%	
	**Total**	**64.0**%	**20.0**%	**16.0**%	**100.0**%	
Other	Education	43.3%	23.3%	33.3%	100.0%	**0.000**
	Architecture	60.0%	24.0%	16.0%	100.0%	
	Engineering	50.0%	25.0%	25.0%	100.0%	
	Theology	40.0%	26.6%	33.4%	100.0%	
	**Total**	**46.6**%	**24.0**%	**29.3**%	**100.0**%	

In the evaluation of the extent to which cycling habits are affected by climatic conditions, it was found that the most intensive cycling occurred on hot and sunny days (Table 22). Therefore, climatic conditions are regarded as a very important factor for bicycle transportation.

**Table 22:** Bicycle use according to climatic conditions.

How Often Do You Use Bicycles According to Climatic Conditions?				
Values	I Use	I Am Undecided	I Do Not Use	Total
	%	%	%	%
Rainy days	28.0%	34.6%	37.3%	100.0%
On cold days	34.6%	28.0%	37.3%	100.0%
Snowy days	6.0%	14.0%	80.0%	100.0%
Foggy days	5.3%	10.0%	84.6%	100.0%
Sunny days	90.0%	6.6%	3.3%	100.0%

In the comparative analysis conducted to determine the effect of cultural values and habits on cycling, a significant relationship was found (p = 0.000). Specifically, 80.0% of those who stated that they did not use bicycles due to social pressure were studying at the Faculty of Theology, while 75.0% of those studying at the Faculty of Engineering were undecided about this situation. Among those who stated that they did not use bicycles due to adverse climatic conditions, 46.6% were studying at the Faculty of Architecture (Table 23).

**Table 23:** The stated reasons related to cultural values and habits contributing to the non-use of bicycles according to the faculty of study.

What Are the Reasons for Not Using Bicycles Related to Cultural Values and Habits?						
Values	Faculties	I agree	Undecided	Disagree	Total	*p*
		%	%	%	%	
In case of public shame (social pressure)	Education	43.3%	22.2%	34.4%	100.0%	**0.000**
	Architecture	20.0%	46.6%	33.3%	100.0%	
	Engineering	10.0%	75.0%	15.0%	100.0%	
	Theology	80.0%	8.0%	12.0%	100.0%	
	**Total**	**42.6**%	**29.3**%	**28.0**%	**100.0**%	
Due to unfavorable climatic conditions	Education	38.8%	22.2%	38.8%	100.0%	**0.001**
	Architecture	46.6%	20.0%	33.3%	100.0%	
	Engineering	40.0%	35.0%	25.0%	100.0%	
	Theology	32.0%	32.0%	36.0%	100.0%	
	**Total**	**38.6**%	**25.3**%	**36.0**%	**100.0**%	

In the evaluation of the transportation modes preferred by students who do not use bicycles, it was determined that these students frequently prefer to use public transportation or walk (Table 24).

**Table 24:** Type of transportation preferred by non-cyclists for their daily trips.

What Is the Type of Transportation You Use for Your Daily Journeys and How Often Do You Use it?				
Values	Frequently	Sometimes	Very Rare	Total
	%	%	%	%
Pedestrian	48.6%	34.6%	16.6%	100.0%
Automobile	4.6%	18.0%	77.3%	100.0%
Bus/minibus	57.3%	36.0%	9.3%	100.0%
Other	22.0%	30.0%	48.0%	100.0%

In the comparative analysis of social pressure and cultural values and the gender of those respondents who do not use bicycles, the proportion of students who stated that they did not use bicycles due to social pressure was higher among males than females. Male respondents avoid using bicycles for reasons such as being humiliated and looked down upon by society (Table 25).

**Table 25:** Reasons related to cultural values and habits that contribute to not using bicycles by gender.

What Are the Reasons for Not Using Bicycles Related to Cultural Values and Habits?						
Values	Gender	I Agree	Undecided	Disagree	Total	*p*
		%	%	%	%	
I don’t know how to ride a bike	Woman	37.8%	4.8%	57.2%	100.0%	**0.002**
	Man	21.2%	4.2%	74.4%	100.0%	
	**Total**	**32.6**%	**4.6**%	**62.6**%	**100.0**%	
I don’t have a bicycle	Woman	23.3%	0.0%	76.6%	100.0%	**0.001**
	Man	21.2%	0.0%	78.7%	100.0%	
	**Total**	**22.6**%	**0.0**%	**78.6**%	**100.0**%	
In case of public shame (social pressure)	Woman	55.3%	9.7%	40.7%	100.0%	**0.000**
	Man	78.7%	4.8%	4.8%	100.0%	
	**Total**	**62.7**%	**10.0**%	**31.3**%	**100.0**%	

These findings are consistent with earlier studies showing that students from faculties with curricula integrating sustainability (e.g., Architecture, Education) demonstrate higher awareness of the social dimension of transportation [70]. Similarly, Van Acker et al. (2010) emphasized that travel behavior is strongly mediated by educational and social-psychological factors [30]. This study thus reinforces the argument that vocational education contributes to shaping sustainable mobility attitudes.

In the comparative analysis of the respondents who do not use bicycles because they do not know how to ride one, do not own one, or do not like cycling, no significant relationship was found with the faculty of study (p > 0.05) and this was not considered further.

These findings illustrate how different social dimension indicators reflect the cognitive, affective, and behavioral aspects of students’ awareness. For instance, equity perceptions relate to cognitive understanding, health and safety attitudes reflect affective awareness, and cycling habits demonstrate behavioral awareness.

## 4. Evaluation and discussion

The evaluation and discussion of the findings were structured around the main and sub-research questions defined in the methodology section. The analysis was conducted through six core social dimension indicators that constitute the framework of sustainable transportation. These indicators enabled a comprehensive assessment of university students’ awareness of the social dimension of sustainable transportation.

In the context of sustainable mobility research, the social dimension of transportation has increasingly gained importance as it addresses issues such as accessibility, equity, social inclusion, and quality of life within urban mobility systems. Recent studies emphasize that sustainable transportation systems should not only minimize environmental impacts but also promote socially inclusive mobility opportunities for different social groups [10,11,15]. Evaluating bicycle use through indicators such as equality, accessibility, health, and cultural values therefore provides an important analytical framework for understanding how mobility practices contribute to socially sustainable urban systems.

The findings indicate that awareness of the social dimension of sustainable transportation among university students is shaped not only by socio-demographic characteristics but also by educational background, spatial conditions, and socio-cultural factors. Significant relationships were identified between gender and perceptions of health and safety, income level and accessibility awareness, and cultural awareness and the equity indicator. These results suggest that the indicators representing the social dimension of sustainable transportation are interrelated rather than independent.

This interconnected structure is also highlighted in the literature on socially sustainable mobility, which suggests that transport systems should be evaluated as complex socio-technical systems where behavioral motivations, spatial accessibility, and social values interact to shape travel behavior [14,15].

In this context, the following discussion examines how these indicators interact with students’ educational backgrounds and socio-demographic characteristics to shape their perceptions and behaviors related to sustainable mobility.

### 4.1. Equality ındicator

The findings revealed that female students used bicycles more frequently than male students (60%), indicating a higher level of awareness among women regarding equality in transportation. This difference may be associated with the higher sensitivity of female students to environmental and social sustainability issues, which has been highlighted in previous research on pro-environmental behavior. In addition, cycling may represent a more accessible and flexible mobility option for female students in urban environments where transportation resources are limited.

Income differences also influenced bicycle use, as most cyclists belonged to middle- and low-income groups (73.2%). This finding suggests that bicycles are often perceived as a practical and affordable transportation alternative for students with limited financial resources. Similar patterns have been reported in previous studies, which emphasize that socio-demographic characteristics and value orientations play an important role in shaping sustainable mobility behavior [29].

From the perspective of social sustainability, the equality indicator highlights the importance of ensuring fair and inclusive mobility opportunities for different social groups. Cycling can therefore be considered not only an environmentally friendly transport mode but also a socially inclusive mobility option that may reduce transport-related inequalities among students [15,71].

### 4.2. Accessibility ındicator

Approximately 47.3% of participants reported using bicycles for short distances. Education Faculty students primarily cycled distances under 3 km (51.1%), while Architecture and Engineering students used bicycles more for medium distances. These differences may be explained by the spatial distribution of faculties within the city and the daily mobility patterns required by different academic programs.

In particular, faculties located closer to residential areas or the city center appear to encourage short-distance cycling, whereas faculties located farther from student housing areas require longer travel distances. Previous studies on sustainable mobility have shown that spatial proximity and urban form significantly influence travel behavior and the adoption of active transportation modes [70].

Recent urban mobility research also emphasizes that compact urban form, mixed land use, and spatial proximity between daily activity locations can significantly increase the likelihood of choosing active transportation modes such as cycling and walking [11].

In this context, improving spatial accessibility between university campuses, residential areas, and city centers is essential for increasing bicycle use.

### 4.3. Health and safety ındicator

About 77.4% of the students stated that they cycled for health benefits, indicating that physical well-being is a major motivation influencing transportation choices. This finding highlights the importance of health-related motivations in promoting active mobility among young populations.

From a behavioral transportation perspective, such motivations can be interpreted within the framework of the Theory of Planned Behavior [72], which suggests that individuals’ behavioral intentions are shaped by attitudes, subjective norms, and perceived behavioral control. In this context, positive attitudes toward health benefits and physical well-being may strengthen students’ intentions to adopt cycling as a preferred mode of transport.

However, Architecture students reported more frequent conflicts with pedestrians (72%), highlighting perceived safety concerns in shared urban spaces. Such conflicts may arise from the lack of clearly defined bicycle lanes or insufficient separation between pedestrian and cycling areas. Perceived safety has been widely recognized as a key determinant of cycling behavior, as individuals are less likely to adopt cycling if they perceive the urban environment as unsafe [67].

Recent studies on cycling behavior also confirm that perceived safety and infrastructure quality are among the most influential factors shaping individuals’ willingness to use bicycles in urban environments [11,15].

Therefore, improving infrastructure design and ensuring safer shared spaces are critical for encouraging bicycle use.

### 4.4. Individual responsibility ındicator

Overall, 52.6% of participants preferred bicycles for economic reasons and 46% for environmental benefits. Notable faculty-based differences emerged: 80% of Architecture students cited environmental motives, whereas 60% of Engineering students emphasized economic savings.

These differences may reflect variations in educational perspectives and disciplinary orientations. Students in design-oriented programs such as architecture may be more exposed to discussions on sustainability, environmental design, and urban ecology, which may influence their environmental awareness. Conversely, engineering students may evaluate transportation choices more in terms of efficiency and economic practicality.

These results also support behavioral mobility research suggesting that individuals’ travel choices are influenced by different value orientations, including biospheric values related to environmental responsibility and egoistic values associated with economic benefits [10,62].

### 4.5. Integrated planning ındicator

Only 17.3% of respondents reported combining bicycles with other modes of transport. Architecture students showed the highest multimodal use (28%), mostly in combination with public buses. The limited integration of bicycles with other transportation modes suggests that the existing transportation system in Amasya does not sufficiently support multimodal mobility.

From a sustainable urban mobility perspective, integrating cycling with public transportation systems is widely considered a key component of effective transportation planning. Multimodal systems allow travelers to combine different transportation modes efficiently, thereby increasing the attractiveness and feasibility of sustainable travel options [11,38].

This situation reflects a broader challenge frequently identified in medium-sized cities, where transportation planning tends to prioritize motorized travel rather than integrated mobility systems.

### 4.6. Cultural values and habits ındicator

Socio-cultural pressures and climatic conditions strongly influenced bicycle habits among university students. For instance, 80% of Theology students reported avoiding cycling due to social pressure, while Architecture students displayed stronger pro-cycling attitudes.

The influence of socio-cultural factors on travel behavior has been widely documented in mobility studies, which highlight the role of social norms, habits, and perceptions in shaping transportation preferences [30].

Behavioral transportation theories also emphasize that social norms and collective perceptions play a crucial role in shaping mobility behavior. According to the Theory of Planned Behavior, perceived social expectations and cultural attitudes may significantly influence individuals’ willingness to adopt sustainable transportation modes such as cycling.

In addition, climatic conditions also affected cycling habits. Approximately 90% of users reported cycling on sunny days, while more than 80% avoided cycling during snow or fog. These findings indicate that both environmental and socio-cultural factors play an important role in determining cycling behavior. Therefore, increasing social awareness and fostering positive societal attitudes toward bicycle transportation may contribute to the wider adoption of sustainable mobility practices in university environments.

Overall, the analysis indicates that awareness levels vary significantly according to faculty affiliation and socio-demographic characteristics. Architecture and Education students demonstrated higher cultural and environmental awareness, whereas Engineering students tended to emphasize economic considerations and Theology students were more influenced by social norms. These differences suggest that educational background plays an important role in shaping awareness of the social dimension of sustainable transportation.

The observed differences between faculties indicate that sustainable mobility awareness is not shaped solely by individual preferences but is also influenced by educational environments and disciplinary perspectives. Educational programs may expose students to different conceptual frameworks related to sustainability, urban development, and environmental responsibility. For example, architecture and planning-related fields often emphasize environmental design and sustainability concepts, which may contribute to higher levels of environmental awareness among students in these disciplines. In contrast, students in technically oriented fields such as engineering may approach transportation choices from a more functional and economic perspective, while socio-cultural norms and collective expectations may influence mobility behavior in certain social groups, as observed among theology students.

These findings are consistent with studies conducted in other university cities, which demonstrate that students’ mobility behavior is shaped by a combination of environmental awareness, economic considerations, and social norms. Previous research has also shown that cycling behavior in university settings is influenced not only by the availability of infrastructure but also by cultural attitudes toward bicycle use and perceived safety conditions. Therefore, the patterns observed in Amasya reflect broader trends identified in the literature.

From a policy and planning perspective, these findings highlight the importance of integrating the social dimension of sustainable transportation into university mobility strategies. Promoting bicycle use should involve not only improvements in physical infrastructure but also educational initiatives and awareness programs that address social perceptions and cultural attitudes toward sustainable mobility. In this context, collaboration between university administrations and local governments can play a crucial role in developing integrated mobility strategies that encourage sustainable travel habits among students.

Despite its contributions, this study has several limitations that should be acknowledged. First, the empirical data were collected exclusively from students of Amasya University within the specific urban context of the city of Amasya. The geographical characteristics, topographic structure, urban scale, transportation infrastructure, and socio-cultural dynamics of Amasya differ from those of many other university cities in Türkiye and internationally. Therefore, the findings should be interpreted within the specific context of Amasya and cannot be directly generalized to all university cities. Nevertheless, the study provides valuable insights into how awareness of the social dimension of sustainable transportation may develop within a university environment. Despite this limitation, Amasya represents a meaningful case for examining student awareness due to its relatively compact urban structure and the presence of bicycle infrastructure within the university environment.

A second limitation concerns the scope of the sample. The research was conducted with students from four faculties—Education, Architecture, Engineering, and Theology—which play an important role in representing the academic structure of the university. However, the exclusion of other academic fields such as health sciences, economics, and social sciences may limit the ability of the study to fully capture the diversity of awareness related to sustainable transportation across different educational backgrounds. Future studies including students from a broader range of academic disciplines and conducted in different university cities would contribute to a more comprehensive understanding of how educational background and urban context influence awareness of the social dimension of sustainable transportation.

## 5. Conclusions and recommendations

This study examined university students’ awareness of the social dimension of sustainable transportation, focusing on bicycle use as a key component of sustainable mobility. Beyond identifying awareness levels, the study also aimed to understand how educational background, social perceptions, and spatial conditions influence students’ attitudes toward bicycle use. The conclusions and recommendations were structured to address the main and sub-research questions.

### 5.1. Answers to the main research questions

- The findings reveal a significant relationship between university students’ awareness levels of the social dimension of sustainable transportation and the six key indicators of social sustainability (equity, accessibility, health and safety, individual responsibility, integrated planning, and cultural values and habits). Awareness was defined as a multidimensional construct encompassing cognitive, affective, and behavioral components, effectively measured through attitude analysis.
- The vocational education processes in different faculties were found to influence students’ awareness levels in distinct ways. The Faculties of Architecture and Education exhibited higher awareness compared to the Faculties of Engineering and Theology. This difference can partly be explained by the curricular structure of these faculties, where topics such as sustainability, environmental responsibility, and social awareness are more frequently integrated into educational programs. As a result, students in these faculties may develop stronger sensitivity toward sustainable mobility issues.

Awareness levels were positively correlated with favorable attitudes toward bicycle use, particularly in relation to health, environmental, and equity dimensions. This finding suggests that increasing awareness of the social dimension of sustainable transportation can contribute to strengthening students’ willingness to adopt cycling as a sustainable transportation alternative.

### 5.2. Conclusions

The general evaluation of the analyses conducted in this study indicates that students in the Faculties of Architecture and Education experience problems related to accessibility and integrated planning principles, while students in the Faculties of Theology and Engineering face challenges associated with cultural values, habits, and equality principles.

These differences indicate that sustainable transportation awareness is influenced not only by physical infrastructure but also by educational context and social perceptions. Faculties that include environmental planning, spatial design, or social awareness topics within their curricula tend to promote a higher level of sensitivity toward sustainability-related issues.

Additionally, the sense of individual responsibility, one of the social dimension principles of sustainable transportation, was found to be higher among students in the Faculties of Architecture and Education compared to students from other faculties. Therefore, the inclusion of social and sustainability-related courses in the curricula of faculties such as Architecture and Education contributes to the development of students’ awareness levels.

The findings indicate that faculty affiliation strongly shapes awareness of the social dimension of sustainable transportation. Architecture and Education students showed higher sensitivity to cultural and environmental factors, while Engineering students emphasized economic and practical aspects. These results suggest that disciplinary perspectives influence how students interpret transportation alternatives and evaluate sustainable mobility options such as cycling.

Another important finding concerns the role of social perceptions in shaping transportation behavior. The factor described as “social pressure” in this study reflects the influence of peer groups and social norms on bicycle use. In some contexts, bicycles may still be perceived as vehicles associated with lower-income groups or as recreational tools rather than legitimate transportation modes. Such perceptions may discourage students from adopting cycling as a daily mobility practice.

These findings are consistent with previous studies conducted in different cities, which demonstrate that the success of cycling policies depends not only on infrastructure development but also on transforming social attitudes toward sustainable mobility.

### 5.3. Recommendations

The following recommendations have been developed to increase bicycle use in universities by enhancing awareness of the social dimension of sustainable transportation. These recommendations are derived directly from the empirical findings of the study and aim to address the social, spatial, and institutional factors influencing bicycle use among university students.

According to the principle of ***equity:***

- Social activities should be organized particularly for male students to emphasize that cycling does not lead to a loss of social status but rather serves as a tool for raising awareness.
- Bicycle festivals organized within faculties should be open to the participation of all individuals and civil society organizations without gender discrimination.
- To change the perception that “bicycles are vehicles for low-income groups,” joint bicycle festivals should be held with the participation of university administrators, faculty members, students, high-income groups, and socially recognized figures.

According to the principle of ***accessibility***:

- The continuity of existing bicycle paths should be ensured.
- New bicycle paths providing safe access from the university campus to the city center should be constructed.
- Special attention should be given to improving spatial connectivity between campus areas and urban centers, as accessibility significantly influences students’ decisions to use bicycles as a transportation mode.

According to the principle of ***health and safety:***

- Training sessions should be organized to raise awareness among motor vehicle drivers about bicycle transportation and to address cyclists’ safety concerns.
- Appropriate infrastructure facilities should be established to ensure the safety of bicycle users.
- At intersections with mixed traffic, bicycles should be given priority through horizontal and vertical markings and signaling systems.
- Adequate lighting should be provided on bicycle paths, especially at night.
- Educational programs should be conducted to explain the positive impacts of cycling on both economic and physical/psychological health.
- Large-scale festivals and social events should be held on campus to promote the health benefits of cycling and to foster societal acceptance.

According to the principle of ***integrated transportation:***

- Fare policies accommodating bicycle users on public transport should be developed, and bicycles should be included in the electronic card system.
- Arrangements should be made for transporting bicycles inside or outside public transportation vehicles.
- Land use and transportation plans should be integrated, considering pedestrian and bicycle access distances in public space planning.
- Continuous bicycle paths and parking areas should be created near educational facilities.
- Cooperation between university administrations and local governments should be established to build safe bicycle routes within campuses.
- Bicycles should be integrated into smart transportation systems, and these systems should be publicly promoted to increase awareness.
- Projects facilitating access to shared on-campus areas should be supported to strengthen social cohesion among students.

According to the principle of ***individual responsibility:***

- Courses focusing on sustainable transportation, especially on its social dimension, should be included in all university curricula.
- It should be emphasized that cycling contributes to sustainable urbanization not only as a sport or social activity but also as a preferred mode of transportation for accessibility.
- University leaders (rectors, deans, etc.) should act as role models by actively using bicycles.

According to the principle of ***cultural values and habits:***

- Seminars should be organized at universities to highlight the contribution of bicycle transportation to social development.
- To reduce social pressure against cycling, public bicycle-sharing projects that ensure integration between campus areas should be expanded.
- To foster cycling habits, incentive and reward programs should be implemented to encourage students to use bicycles regularly.

Overall, promoting bicycle use requires a comprehensive approach that combines infrastructure development, educational initiatives, and social awareness strategies.

To promote the widespread use of bicycles across the city and to achieve sustainable urban development, it is essential to raise the awareness levels of the younger population—particularly university students. In this regard, courses emphasizing sustainable transportation and its social dimension should be incorporated into the curricula of all higher education programs.

The study conducted in the city of Amasya presents recommendations that are applicable to other cities and contribute significantly to sustainable urban development.

In conclusion, enhancing awareness of the social dimension of sustainable transportation, when supported by physical planning, public participation, and community-based awareness initiatives, will make a substantial contribution to achieving sustainable and bicycle-oriented urbanization.

**Institutional Review Board Statement:** Ethic Committee Name: Ondokuz Mayıs Üniversitesi, Sosyal ve Beşeri Bilimler Araştırmaları etik Kurulu (Ondokuz Mayıs University, Social and Human Sciences Research Ethics Committee), Approval Code: 2025−300, Approval Date: 28.03.2025

**Informed Consent Statement:** Informed consent was obtained from all individuals who participated in the study.
